# Tobacco Cessation Quitline Spending in 2005 and 2006: What State-Level Factors Matter?

**DOI:** 10.3390/ijerph6010259

**Published:** 2009-01-13

**Authors:** Paula A. Keller, Eric J. Beyer, Timothy B. Baker, Linda A. Bailey, Michael C. Fiore

**Affiliations:** 1 University of Wisconsin Center for Tobacco Research and Intervention, University of Wisconsin School of Medicine and Public Health, 1930 Monroe Street, Suite 200, Madison, Wisconsin 53711, USA; E-Mails: ejbeyer@ctri.medicine.wisc.edu (E. J. B.); tbb@ctri.medicine.wisc.edu (T. B. B.); mcf@ctri.medicine.wisc.edu (M. C. F.); 2 North American Quitline Consortium, 3030 N Central Avenue, Suite 602, Phoenix, Arizona, 85012-2713, USA; E-Mail: lbailey@naquitline.org

**Keywords:** Smoking, Tobacco Use Cessation, Quitlines, State Funding, Tobacco Control Policy

## Abstract

Tobacco cessation telephone quitlines are an effective population-wide strategy for smoking cessation, but funding for this service varies widely. State-level factors may explain this difference. Data from the 2005 and 2006 North American Quitline Consortium surveys and from publicly available sources were analyzed to identify factors that predict higher levels of *per capita* quitline funding. The best-fitting multivariate model comprised higher *per capita* tobacco control funding (2005 p = 0.004, 2006 p=0.000), not securitizing Master Settlement Agreement payments (2005 p = 0.008, 2006 p=0.01), and liberal political ideology (2005 p = 0.002, 2006 p=0.002). Select state-level factors appear to have influenced *per capita* quitline services funding. These findings can help inform advocates and policymakers as they advocate for quitlines and tobacco control funding.

## 1. Introduction

Tobacco cessation quitlines have been shown to be an efficacious, effective and cost-effective population-wide strategy for smoking cessation [1–5]. All 50 states, the District of Columbia, Puerto Rico, and many other countries now have quitlines in place, providing evidence-based counseling and in some instances, medications to callers [6, 7].

Despite the evidence base supporting the effectiveness of quitlines, recommendations for inclusion of comprehensive quitline services as part of state tobacco control programs, and increased targeting of quitlines by state and national tobacco control efforts [8–10], the amount of funding available for quitline services varies widely. An analysis of U.S. quitline services budgets in 2004 found that the median quitline services budget was $500,000 (range $40,000–$3,800,000) [11]. Analyses of U.S. quitline services budgets in 2005 and 2006 found similar ranges of funding [6]. In contrast, in 2007 the U.S. Centers for Disease Control and Prevention recommended significant increases in state spending for tobacco control programs, with recommended funding levels for cessation services (including but not limited to quitlines) ranging from $1.9 million to $103.7 million [8].

Few researchers have attempted to link state characteristics (state-level factors) with funding for either tobacco control programs or for quitlines, and findings from previous research have been mixed [12–15]. Factors that have been evaluated in these studies include state demographic variables, *per capita* tobacco control spending, smoking prevalence rates, amount of tobacco produced, state political ideology, and political affiliation of governors and legislatures. The current study was designed to expand our previous work to identify state-level factors that may influence quitline funding. A more comprehensive understanding of such factors has the potential to aid advocates and policymakers in more effectively advocating for quitlines and tobacco control programs.

## 2. Methods

### 2.1. Variables and Data Sources

Self-reported quitline services budgets from the 2005 and 2006 North American Quitline Consortium’s (NAQC) survey of state quitlines and total population estimates from the U.S. Census Bureau were used to create our dependent variables, *per capita* quitline services budget for 2005 and 2006. The 2005 and 2006 NAQC surveys are the most recent surveys for which data are available. The NAQC survey methodology has been published elsewhere [6]. States were instructed to report all sources of funding (e.g., state, federal, non-governmental) when reporting the budget for quitline services for the state quitline. States were instructed to report quitline promotional spending separately from quitline services spending. Promotional funds were not included in this analysis.

State-level factors were identified from publicly available data sources and used as independent variables in our analysis. A detailed description of the methodology and the data sources has been published elsewhere [15]. Demographic variables (median age, median income, percent of the population with at least a high school education) were obtained from the U.S. Census Bureau [16, 17]. Tobacco use variables (adult smoking prevalence, cigarette consumption) were obtained from the Centers for Disease Control and Prevention and the Tax Burden on Tobacco [18–20]. Tobacco control spending variables (*per capita* tobacco control expenditures, whether a state securitized any or all of its Master Settlement Agreement payments) were obtained from the Campaign for Tobacco-Free Kids and the U.S. Census Bureau [16, 17, 21]. Political and economic climate measures [cigarette excise tax, state political ideology (defined on a 0–100 scale, with 0 = “conservative” and 100 = “liberal”), the political affiliation of the governor (defined as “Republican” or “Democrat”), the political affiliation of the legislature (defined as percent Republican), state budget deficit, and tobacco production greater than or equal to 1 million pounds] were obtained from publicly available reports, the peer-reviewed literature, the National Governor’s Association, the National Conference of State Legislatures, the Center on Budget Policy and Priorities, and the United States Department of Agriculture [20, 22–29].

The University of Wisconsin Health Sciences Institutional Review Board (IRB) determined that the study protocol was exempt from IRB review.

### 2.2. Analyses

All variables were reviewed for distributional problems such as skewness or outliers. Data with distributional problems were transformed to permit regression analysis. Median split transformations were used for the state budget deficit and *per capita* tobacco control expenditure variables. Outliers were moved within three standard deviations of the mean, preserving the order of the data, for the cigarette consumption variable. All data were re-inspected to ensure the distributional problems had been corrected and that the order of the data had been preserved prior to analysis. Univariate and multivariate linear regression analyses were conducted using SPSS to identify potential predictors of *per capita* quitline budgets in 2005 and 2006 (SPSS, version 15).

Univariate results were considered statistically significant at p ≤ 0.05. Variables that were significant at p ≤ 0.25 in the univariate analysis were included in the multivariate analyses. Backwards model building techniques described by Hosmer and Lemeshow were utilized [30]. Variables were removed one at a time from the model until all remaining variables were significant at p ≤ 0.05.

## 3. Results

Table 1 summarizes the univariate results for 2005 and 2006. In the univariate analysis, *per capita* tobacco control expenditures and political ideology were statistically significant in 2005. These variables were also statistically significant in 2006, with the addition of education and tobacco production. In both 2005 and 2006, additional variables approached but did not reach statistical significance (median age, MSA securitization, and cigarette excise tax rate).

The following variables were included in the 2005 multivariate analysis: *per capita* tobacco control expenditures, master settlement agreement securitization, political ideology, tobacco production, governor’s political affiliation, and median age.

Three variables were significant in the final multivariate model:

- master settlement agreement securitization [p=0.008, b=–0.243, SE (95% CI) =–0.417 to –0.068]
- *per capita* tobacco control expenditures [p=0.004, b=0.254, SE (95% CI) =0.089 to 0.420]
- political ideology [p=0.002, b=0.007, SE (95% CI) =0.003 to 0.011].

The 2006 multivariate analysis comprised the following variables: *per capita* tobacco control expenditures, master settlement agreement securitization, political ideology, tobacco production, cigarette excise tax rate, and education. As in 2005, the following variables were significant in the final multivariate model:

- master settlement agreement securitization [p=0.010, b= –0.205, SE (95% CI) = –0.359 to –0.052]
- *per capita* tobacco control expenditures [p=0.000, b=0.334, SE (95% CI) =0.191 to 0.478]
- political ideology [p= 0.002, b= 0.006, SE (95% CI) =0.002 to 0.009].

## 4. Discussion

These findings represent state-level factors that may influence *per capita* quitline funding. *Per capita* tobacco control spending and political ideology were the only variables that were statistically significant in both the univariate and multivariate analyses. Whether a state had securitized part or all of its MSA payment was also statistically significant in the multivariate analysis.

By 2006, all 50 US states, the District of Columbia, and Puerto Rico had implemented quitlines as part of their comprehensive tobacco control programs, consistent with CDC recommendations [8]. It is reasonable to infer that states with well-funded tobacco control programs would also fund their quitlines at higher levels *per capita*. The positive relation between *per capita* tobacco control spending and *per capita* quitline spending is also consistent with our previous research [15].

Whether a state securitized any or all of its MSA payment had a negative relation with *per capita* quitline spending. Tobacco control advocates have long been concerned that MSA securitization would have a negative impact on state tobacco control programs. However, we were unable to find any published analyses in this area. Our results suggest the advocates’ concerns are valid, but further analysis of trends in securitization and allocations of MSA payments for tobacco control at the state level is warranted to confirm this finding.

We also found that *per capita* quitline service budgets were predicted by a state’s political ideology in both our univariate and multivariate analysis. That is, states with a more liberal ideology spent more *per capita* on quitline services than states with a more conservative ideology. The literature in this area is mixed. Snyder *et al*. did not find political ideology to be influential in their analysis of state-level factors affecting funding allocations for tobacco control programs [12]. Similarly, our prior analysis did not find political ideology to be a factor in predicting the amount of *per capita* spending on quitlines [15]. Sloan and colleagues did not find political ideology to be influential in their analysis of total *per capita* MSA spending, but did find that states with a more liberal ideology were less likely to securitize their MSA payments [14]. However, a longitudinal analysis by Blais and colleagues found that governments controlled by the majority party and with a more liberal ideology tended to spend slightly more than governments with a more conservative ideology, but the differences were relatively modest (4% increase over six years) [31]. A possible explanation of the differences in our findings between the work of Snyder and Sloan is the difference in time period evaluated (Snyder – 2001, Sloan – 2000–2003). Our study also used a more recently published measure of political ideology than did Snyder and Sloan. Finally, our current analysis comprised all 50 state quitlines in the US, the District of Columbia, and Puerto Rico; our previous analysis only comprised the 38 states with quitlines in 2004. Our findings suggest that political ideology may play a role in determining *per capita* quitline service budgets, but additional research is required to verify this finding.

Limitations of our analysis include the use of self-reported quitline budget data to construct our dependent variable and use of publicly reported data on our independent variables. Some states have extensive private-sector funding of quitline services through health insurers or employers, which is not reflected in this analysis of state-funded quitline services. Further, the use of correlational research strategies does not permit strong causal inferences to be made. Finally, it is possible that other unmeasured variables are correlated with *per capita* quitline spending and their inclusion in analyses could affect the obtained prediction models.

Results of this study indicate that state-level factors appear to influence *per capita* quitline services budgets. Findings from this and related research may aid advocates and policymakers in understanding how to more successfully advocate for quitlines and other public health programs. For example, *per capita* tobacco control spending data can be used in comparison with other, comparable states’ spending data to help argue for additional investments to enhance quitline services – or conversely, to maintain existing levels of investment. Additionally, policymakers may wish to utilize these preliminary findings to help demonstrate the potential negative impact of securitization on quitlines if states are considering securitizing MSA payments given the current economic recession. However, further research, particularly regarding trends in MSA securitization and subsequent allocation of resources for comprehensive tobacco control programs including quitlines, is warranted.

## Figures and Tables

**Table 1.** Univariate results, *per capita* quitline services budget, 2005 and 2006.

	2005			2006		
	p-value	B	SE (95% CI)	p-value	B	SE (95% CI)
Demographi c Information						
Education:≥ high school degree	0.894	–4764.816	–76834.791–67305.160	0.025*	0.024	0.003–0.004
Income	0.947	–3.38E–007	0.000–0.000	0.871	7.71E–007	0.000–0.000
Age	0.074	0.037	–0.004–0.077	0.347	0.021	–0.023–0.065
**Tobacco Use**						
Adult smoking prevalence	0.798	–0.004	–0.034–0.027	0.814	–0.003	–0.033–0.026
Consumption	0.412	0.001	–0.002–0.005	0.677	0.001	–0.003–0.004
**Tobacco Control Spending**						
Securitization of MSA Payments	0.105	–0.164	–0.363–0.036	0.090	–0.168	–0.362–0.027
*Per capita* tobacco control expenditures	0.021*	0.226	0.035–0.416	0.000*	0.378	0.222–0.534
**Economic and Political Climate**						
Cigarette Excise Tax Rate	0.459	0.068	–0.116–0.251	0.070	0.142	–0.012–0.297
Political Ideology	0.011*	0.006	0.001–0.011	0.005*	0.007	0.002–0.011
Governor’s Political Affiliation	0.195	–0.125	–0.318–0.067	0.274	–0.106	–0.298–0.068
Legislature’s Political Affiliation	0.413	0.290	–0.418–0.999	0.902	0.043	–0.649–0.734
State Budget Deficit	0.311	–0.101	–0.299–0.098	0.330	0.125	–0.136–0.386
Agriculture: Tobacco Production	0.083	–0.098	–0.210–0.014	0.020*	–0.126	–0.231–0.021

* Statistically significant at p < 0.05
